# Revisiting classical Escherichia coli cell division mutants by whole-genome sequencing

**DOI:** 10.1099/mgen.0.001558

**Published:** 2025-11-04

**Authors:** Elias Dahdouh, Isabel García-Pérez, Diana Soledad Reyes-Zuñagua, Jesús Mingorance, Miguel Vicente

**Affiliations:** 1 Servicio de Microbiología, Hospital Universitario La Paz, IdiPAZ, Paseo de La Castellana 261, 28046 Madrid, Spain; 2 Centro Nacional de Biotecnologia CSIC, Calle Darwin 3, 28049 Madrid, Spain

**Keywords:** cell division, *Escherichia coli*, genomic analysis, mutagenesis

## Abstract

Over 60 years ago, researchers started the genetic analysis of bacterial cell division by isolating conditional, temperature-sensitive mutants of essential *Escherichia coli* cell division genes. These early mutants were obtained by mutagenesis with chemical agents that introduced dozens to hundreds of mutations in the bacterial genomes. In this work, we present the complete genome sequences of six of these original mutants on *ftsA*, *ftsZ* and *ftsQ* genes, along with two of the strains used to generate them. The genomes of mutants obtained by exposure to nitrosoguanidine had 100 to 400 mutations. Transducing target alleles into a new strain effectively reduced the number of mutations, but those near the target gene were co-transduced with it. In contrast, a mutant generated by site-directed mutagenesis maintained the genomic background intact. The genomic analysis improves our understanding of these foundational strains, offering insights into the effects of historical mutagenesis techniques. These findings underscore the importance of genomic characterization in ensuring accurate interpretations of experimental results in microbiological research.

**Impact Statement**

*Escherichia coli* has been the primary model for bacterial cell division studies for years, with various mutagenesis methods used to identify essential genes and their functions. We sequenced the complete genomes of six key mutant strains and two parental strains to enhance our knowledge of these foundational tools. Our analysis showed that chemically mutagenized strains carried hundreds of unintended mutations, some of them in other cell division genes, whereas targeted mutagenesis yielded mutants with clean genetic backgrounds.

## Data Summary

The whole-genome sequencing data of all strains in this study have been submitted to the National Center for Biotechnology Information database under the BioProject accession number PRJNA1273085 with SRA accession numbers SRR33858938 to SRR33858947 and genome assembly accession numbers CP195014 (MC1061) and CP195020 (OV2).

## Introduction

Cell division is an essential phase of the bacterial cell cycle whose details are still being worked out. The genetic analysis of bacterial cell division started in the 1960s when researchers used *Escherichia coli* to generate conditional mutants of essential cell division genes [1,5]. These were isolated after extensive mutagenesis as *f*ilamentous *t*emperature-*s*ensitive mutants (hence called *fts^-^* mutants), able to grow but not to divide at the restrictive temperature. In this way, several cell division genes were identified, namely, *ftsA*, *ftsB*, *ftsI*, *ftsL*, *ftsQ*, *ftsW* and *ftsZ*. Most of their protein products were later shown to localize at mid cell forming a ring during the division process. Several of these genes were found to map to a small region of the chromosome together with several genes involved in cell wall synthesis. This region was then named the *d*ivision and *c*ell *w*all gene cluster or *dcw* gene cluster [5].

Mutagenesis was often induced by exposure to high concentrations of nitrosoguanidine (N-methyl-N’-nitro-N-nitrosoguanidine), a chemical mutagen that generates a high density of mutations (mainly G:C to A:T transitions) by alkylating the O6 position of guanine and O4 of thymine [6]. Mutants were selected by different methods, for example, by growing the mutagenized cells at a restrictive temperature to induce filamentation, passing the cells through a membrane filter and plating the retained filamentous cells at a lower temperature [7]. The mutations were further isolated and characterized using P1-mediated transduction techniques. These mutants were used to study gene product interactions and identify different steps in the division process [6]. Later, the genes were cloned, sequenced and expressed. The biochemical activities of their purified products were then analysed. GFP fusions and specific antibodies were generated to visualize their location within the division ring [6,7].

The mutants developed and utilized in initial research have historical value because of the foundational insights that they offered (Table 1). The *E. coli* strain OV2 is a temperature-sensitive suppressor mutant (*supF-A81*) obtained from the parental K-12 strain MB93A81 by mutagenesis [8,9]. OV2 was used in studies on cell growth and division because of its consistent growth and morphology under diverse conditions. OV2 was further mutagenized, and selection for cell division mutants yielded the amber mutant *ftsA16* (strain OV16) [10]. Similarly, the *ftsA* mutant alleles *ftsA2* and *ftsA3* had been obtained by nitrosoguanidine mutagenesis [11] from MC-6, a non-K12 strain of environmental origin [12]. They were later transferred to OV2 by P1 transduction, generating the mutant strains D2 [13] and D3 [14,15].

**Table 1.** Origins and relevant phenotypes of the sequenced strains

Strain	Origin	Relevant phenotype	Genome analyses*	References
OV2	Derivative of *E. coli* K-12 strain MB93A81	supF-A81(Ts)	Hybrid assembly	[8]
OV16	Derivative of OV-2	ftsA16am	Mapped to OV2	[10]
D2	ftsA2 mutation of MC-6 transduced to OV-2	ftsA2(ts)	Mapped to OV2	[11,13]
D3	ftsA3 mutation of MC-6 transduced to OV-2	ftsA3(ts)	Mapped to OV2	[11,14, 15]
MC1061	Developed as a cloning vehicle		Hybrid assembly	[19]
VIP205	Engineered derivative of MC1061	Ptac-ftsZ	Mapped to MC1061	[20,21]
PAT84	Derivative of *E. coli* K-12, designated originally as ftsA and renamed later ftsZ84	ftsZ84(ts)		[16,17]
TOE1	ftsQ derivative of AB2497 (derivative of AB1157)	ftsQ(ts)	Mapped to AB1157	[18]

*All eight genomes were analyzed by mapping of short reads against the reference genome MG1655 (U00096.3). The column shows other analyses done.

The strains PAT84 and TOE1 are *ftsZ* and *ftsQ* mutants, respectively, obtained by chemical mutagenesis from K-12 strains. The exact origin and genotype of PAT84 have not been published [16,17]. TOE1 was obtained from AB2497 [18], a derivative of the K-12 AB1157 strain.

The *E. coli* strain MC1061 was developed as a vehicle for cloning by Casadaban and Cohen [19] and was extensively used for cell division and growth studies for its regular growth and morphology. VIP205 is an engineered derivative of MC1061 in which the *ftsZ* gene has been placed under the control of a *lacZ* promoter, being therefore IPTG-dependent for division [20,21].

These division genes and their mutations have already been mapped, cloned and sequenced, but the genomic backgrounds of these heavily manipulated strains have not been studied. These mutants were foundational in the study of bacterial cell division, and their genomes might constitute a valuable historical resource. To gain insight into their genetics, we have sequenced and studied their genomes and compared them with the genomes of their parental strains.

## Methods

### DNA extraction and sequencing

The strains were recovered from −80 °C stocks by plating on LB agar. Single colonies were grown overnight in 2 ml LB broth at 30 or 37 °C, depending on the strain. VIP205 was grown at 37 °C in the presence of 0.02 mM IPTG. Grown cultures were centrifuged and resuspended in 0.5 ml of sterile water, and DNA was extracted in a MagCore^®^ HF16 Plus System automated system (RBC Bioscience Corp., Taiwan). Genomic DNA was fragmented for short-read sequencing using a Covaris M220 ultrasonicator (Covaris Ltd., UK), and libraries were constructed using the NEBNext^®^ Fast DNA Library Prep Set for Ion Torrent^™^ (New England Biolabs^®^, Ipswich, MA, USA). Fragments of 350 bp were selected using an E-Gel^™^ Power Snap Electrophoresis System and sequenced using an Ion Torrent GeneStudio S5^™^ system (Thermo Fisher Scientific^®^, MA, USA). Long-read sequences were obtained using a MinION^™^ Mk1B sequencer (Oxford Nanopore Technologies^®^, UK) using the rapid library preparation kit SQK-RAD004 and Flongle R9.4.1 flowcells.

### Assembly and mapping

All the sequenced genomes were compared to the MG1655 reference genome (U00096.3) by mapping of reads. In addition, hybrid assemblies of OV2 and MC1061 were done with short and long reads and used as references for some comparisons: OV16, D2 and D3 were mapped to the OV2 assembly, and VIP205 was mapped to the MC1061 assembly. TOE1 was mapped to the AB1157 sequenced genome (NZ_CP076404.1). MC1061 and VIP205 were also compared to a previously sequenced MC1061 genome (NZ_CP169569.1). Sequence analyses were done in the European Galaxy server (usegalaxy.eu) [22]. Trim Galore (v 0.6.10) was used for filtering and trimming short reads and Filtlong (v 0.2.1) for long reads. Hybrid assemblies of OV2 and MC1061 were done with Unicycler (v 0.5.1) and polished with Medaka (v 1.7.2) and Polypolish (v 0.6.0) using the long and short reads, respectively. Mapping of short reads to reference genomes was done with minimap2, and variant calling was done with Snippy and Snippy-core (v 4.6.0). Mapping and SNP visualization were done with Proksee [23]. All the raw sequences, as well as the MC1061 and OV2 assemblies, have been deposited in GenBank (BioProject PRJNA1273085). Protein PDB structures and AlphaFold models were searched and visualized in UniProt [24].

## Results

**Fig. 1.**
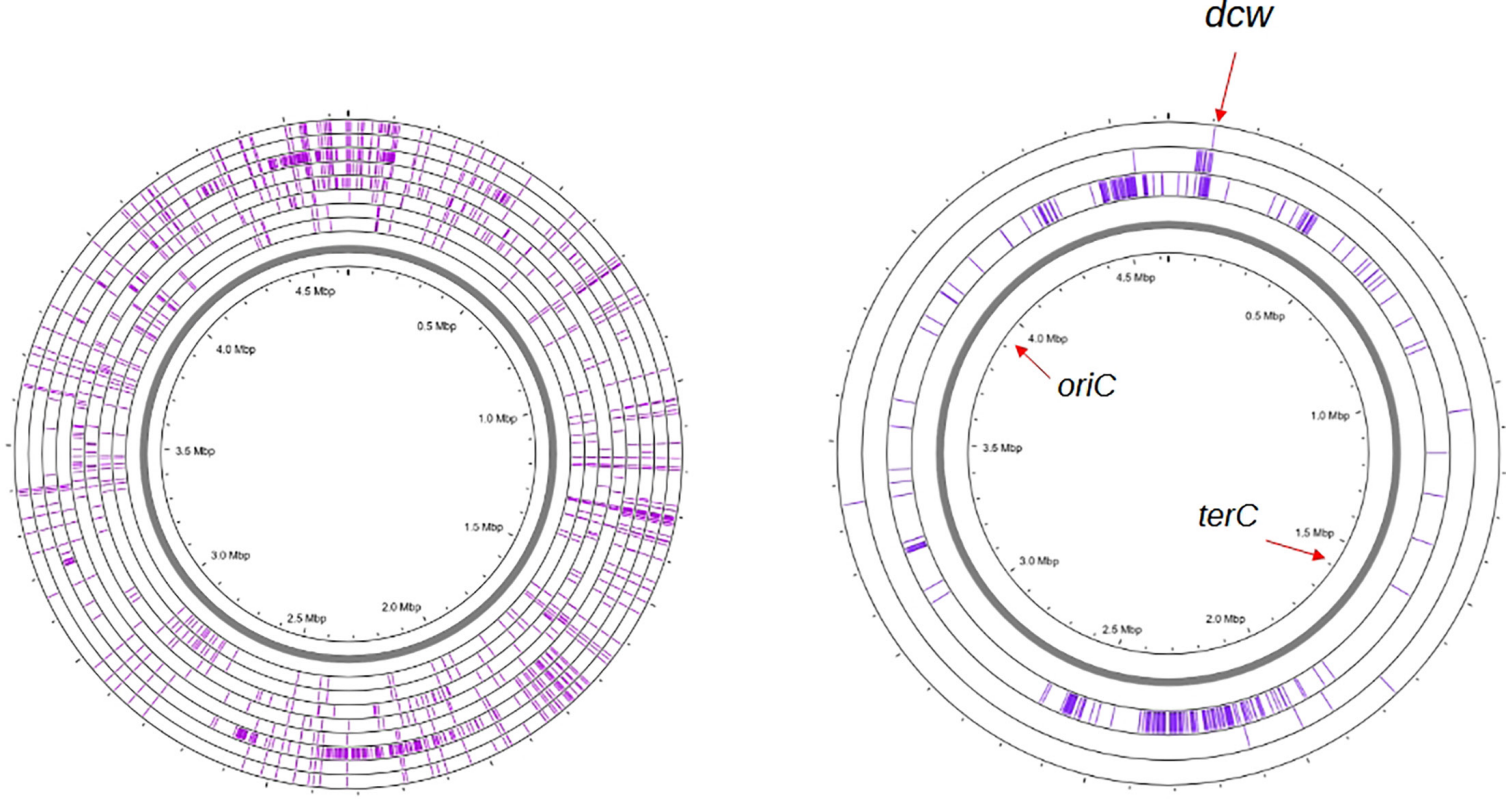
SNP maps of the sequenced strains. (**a**) SNPs identified using MG1655 as the reference genome (grey line), from inner to outer rings: MC1061, VIP205, TOE1, PAT84, OV2, OV16, D2 and D3. (**b**) SNPs remaining after filtering out those common to the four strains: OV2, OV16, D2 and D3; mapped against the OV2 genome (grey line). From inner to outer rings: OV16, D2 and D3.

The genomes of all eight strains were sequenced with short-read sequencing technology. The raw reads were used for variant calling using the *E. coli* MG1655 genome as reference. The core genome analysis detected between 126 and 192 SNPs per strain, except for OV16 that had 442 SNPs (Table 2). On average, 88% of the SNPs were in coding regions, 52% were missense mutations, 28% were synonymous mutations and 4% generated stop codons. SNP distributions in the chromosomes of all eight strains and in the *dcw* cluster are shown in Figs1  2. All the SNP features, including base change, position, evidence and effect, are listed in the Supp. File, available in the online Supplementary File .

**Table 2.** Number of SNPs called by Snippy-core using the short reads of the eight strains and the genome of *E. coli* MG1655 as the reference genome. The percentages indicate the proportion of SNPs found in coding regions, and the proportions of each type of mutation. The complete table, and the tables with all the SNPs listed, are in Supplementary File

Reference: MG1655	SNPs	% in coding regions	% missense mutations	% stop codons	% synonymous mutations
OV2	173	91.3	57.2	5.2	24.9
OV16	442	91.0	55.9	3.8	29.2
D2	179	85.5	55.3	4.5	21.8
D3	192	89.6	59.4	3.6	22.9
PAT84	174	93.7	57.5	2.9	28.2
TOE1	151	85.4	44.4	8.6	25.2
MC1061	126	83.3	43.7	2.4	35.7
VIP205	126	83.3	42.1	4.0	34.9

**Fig. 2.**
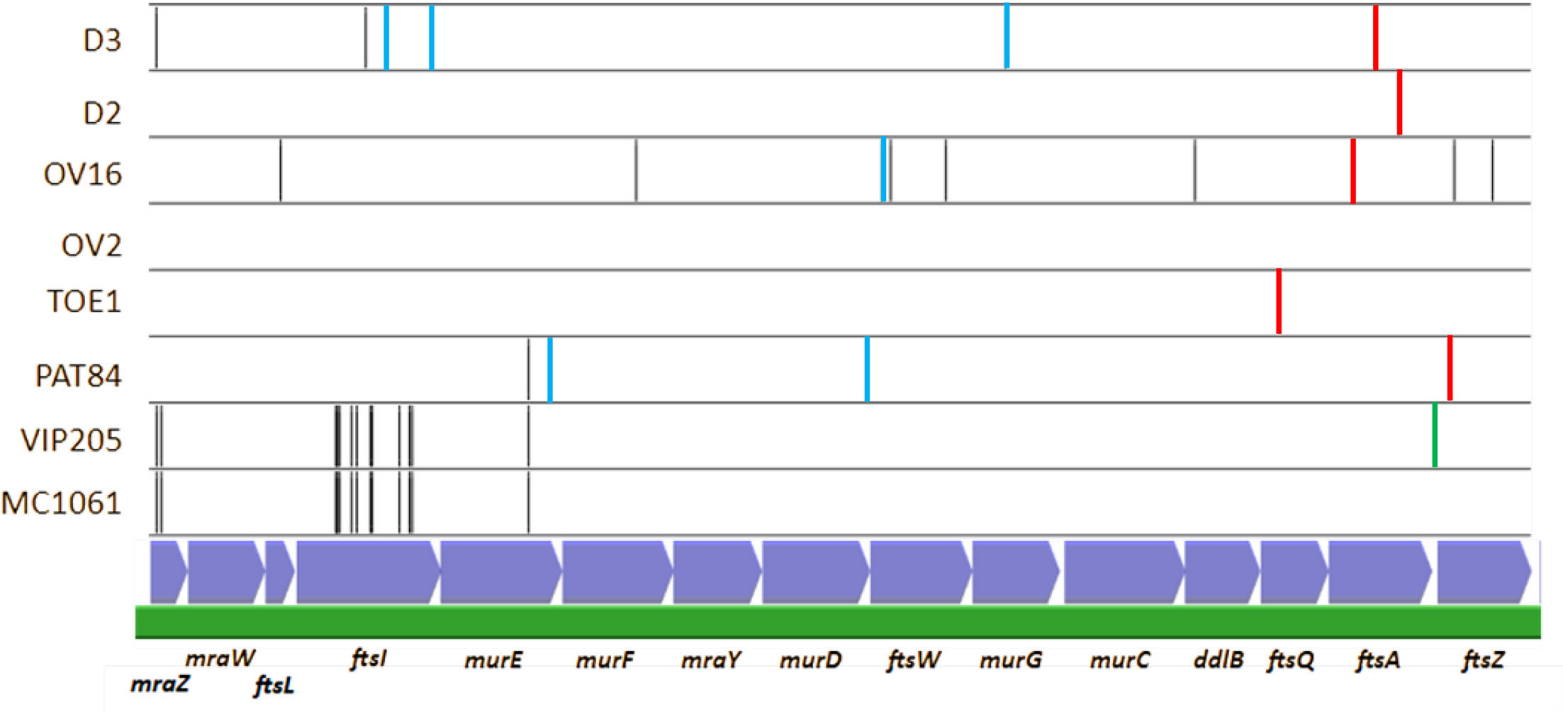
SNP map of the *dcw* gene cluster region in the sequenced strains mapped against MG1655 (green bar and blue arrows). Red vertical lines mark the positions of the mutations responsible for the filamentation phenotype. Blue lines indicate other missense mutations, and black lines are silent mutations. The green line in VIP205 marks the position of the engineered *lacZ* promoter.

Mapping OV2 short reads against MG1655 had a 120× coverage, an 8.5 kb deletion including the *curli* locus and a 15 kb deletion that included a prophage. Variant calling identified 164 SNPs of which 80% were G to A transitions. Mapping OV16, D2 and D3 yielded coverages of 102×, 100× and 127×, respectively. OV16 SNPs were 85% G to A transitions. The three strains had the same deletions as OV2.

The OV2 *supF-A81(Ts)* mutation was detected in the *tyrT* operon. Compared to MG1655, one of the two *tyr* tRNA genes and the non-coding RNA RtT had been lost, and the remaining *tyr* tRNA gene had a C to G mutation that generates a CUA anticodon able to suppress the amber stop codon UAG at the permissive temperature.

Assembly of OV2 short and long reads with Unicycler yielded a single contig that was used as a reference to map its derivatives OV16, D2 and D3. All three strains mapped with a coverage of around 100×. OV16 had 291 SNPs, while D2 and D3 had 13 and 32 SNPs, respectively (Fig. 1b). There were seven SNPs common to all three strains that must have occurred in the OV2-stocked lineage after the *ftsA* mutant selection experiments. There were also five SNPs common to D2 and D3. Filtering out these 12 SNPs left 284, 1 and 20 SNPs in OV16, D2 and D3, respectively. Most of the SNPs in D2 and D3 mapped in the *dcw* gene cluster region and had likely been co-transduced from the mutagenized strains with the *ftsA* mutant genes (Fig. 2).

OV16 had a CAG to UAG amber mutation at codon 155 of the *ftsA* gene. This would result in a Q155Y mutation at the permissive temperature and Q155* at the restrictive temperature. Amino acid residue Q155 is exposed on a β-chain of domain 1C (PDB ID: 7Q6D). This outward-facing location likely explains why the Q155Y mutation doesn't significantly affect FtsA function. In addition, OV16 had several silent mutations in the *ftsL*, *murF*, *ftsW*, *ddlB* and *ftsZ genes* and the missense mutation P78S in FtsW. The residue at position 78 of FtsW is located within a loop of a cytoplasmic domain (PDB ID: 2VH1), and changes in this position are likely to be silent.

D2 had the A338T mutation in FtsA. The A338T mutation is located in an α-helix in domain 2A, near the nucleotide binding site of FtsA, close to the sugar moiety of ATP (PDB ID: 7Q6D). The substitution of alanine (A) with the bulkier threonine (T) at this location may destabilize nucleotide binding, particularly at higher temperature.

D3 had the T240I mutation in FtsA. This position is in an α-helix in domain 2A, and the T residue participates in the binding of the adenine moiety, so the mutation might impair the binding of ATP at high temperatures. While OV16 and D2 are able to resume cell division after being returned to permissive temperature, D3 does not revert, and the reason for this is not known. In addition to the FtsA mutation, D3 had two mutations in FtsI: P372S and R559C. Both residues are situated in the large periplasmic domain. Position P372 lies in an outward-facing loop connecting two helices, while R559 is found in the C-terminal loop (PDB ID: 7ONW). Given their location in exposed loop regions of a non-catalytic domain, these substitutions are unlikely to have a significant phenotypic effect. Finally, MurG had the P170S mutation, located in an outward-facing loop between two helixes in the N-terminal domain (PDB ID: 1F0K). The interaction of FtsA and FtsI occurs through their cytoplasmic domain, while the FtsI mutations are in the periplasmic domain. Besides, MurG is not known to interact directly with FtsA or FtsI. Therefore, we cannot either prove or discard the role of these mutations in the irreversibility of the phenotype, though it seems unlikely.

The short reads of MC1061 had 126 SNPs when compared to MG1655 and 5 SNPs when compared to the Stanford MC1061 genome sequence (GenBank CP169569). A *de novo* assembly of MC1061 was done with short and long reads. The short reads of VIP205 were mapped with Minimap2 against this new reference. In this map, the position of the engineered insertion was detected 28 bp upstream of *ftsZ*, and a single SNP was found with respect to its parental MC1061 strain. The SNP was a missense mutation (T266P) in the carboxy-terminal loop of MepH murein endopeptidase (AlphaFold: AF-P76190-F1), which is likely to be silent.

PAT84 had 174 SNPs with respect to MG1655. Within the *dcw* cluster, there were three missense mutations: G105S in the active site of FtsZ and responsible for the filamentous phenotype. In addition, there were two additional mutations in FtsW and MurE. The FtsW mutation was G15E, in the cytoplasmic loop just before the first transmembrane domain (AlphaFold: AF-P0ABG4-F1). The MurE mutation was V461I in MurE, close to the C-terminus and to the active site cleft in a poorly conserved segment (PDB ID: 1E8C). Both mutations are likely to be silent.

Finally, TOE1 had 151 SNPs with respect to MG1655. When compared to its parental AB1157 genome, a mean coverage of 141× and 43 SNPs distributed through all the genomes were found. The mutant had lost the same prophage lost by OV2, the *motAB-cheAW* operon and part of the type 1 fimbria operon *fim*. The E125K mutation in *ftsQ*, responsible for the cell division phenotype, was detected in the *dcw* cluster. It is part of a β-strand or the turn/loop structure immediately following the α-domain and close to the linker region connecting the N-terminal α-domain to the C-terminal β-domain (PDB ID: 2VH1). The charge change introduced by the mutation might destabilize the relative positions of the two domains, particularly at higher temperatures.

## Discussion

The strains sequenced in this work have been used extensively for bacterial cell division studies for many years. The methods used to generate these strains included mutagenesis with chemical agents known to introduce hundreds of mutations into the chromosome. The most striking example is OV16, which had been generated after two rounds of mutagenesis, one to generate OV2 and another one to generate OV16, and accumulates more than 400 SNPs with respect to MG1655.

The validity of the SNPs detected is supported by three lines of evidence: (1) high-stringency SNP-calling criteria. The default criteria for calling a homozygous SNP are minimum coverage (–mincov) of 10 reads and minimum variant proportion (–minfrac) of 90%. As shown in the Supp. File (Column F, EVIDENCE), the majority of the called SNPs had higher coverage, and in most cases, the alternate allele is present in 100% of the supporting reads. (2) Genetic lineage consistency: when comparing strains belonging to the same lineage (e.g. OV2/OV16/D2/D3 or MC1061/VIP205) to the reference genome (MG1655), the SNPs identified in the parental strains (OV2 and MC1061) were reliably and independently detected in their derivative strains. (3) Correct detection of known mutations: the sequencing successfully identified the known mutations in FtsQ, FtsA and FtsZ.

The development of cloning and site-directed mutagenesis allowed the generation of clean mutants. An example is VIP205, a strain engineered with a clean insertion of a *lacZ* promoter upstream of the *ftsZ* gene [20,21]. Compared with its parental strain, this had a missense mutation in position 266 of the MepH murein endopeptidase, which is far from the *dcw* cluster. Although changes to proline are likely to be disruptive in the protein structure, the position in the carboxy end loop (the protein has 271 residues) and the fact that position 267 is also a proline suggest that this mutation is likely to be neutral. This is further supported by the known functional redundancy of MepH with MepS and MepM endopeptidases [25].

The SNPs were not distributed homogeneously throughout the chromosome; some regions had higher SNP densities (Fig. 1a). This heterogeneity might be the result of asymmetries in DNA replication, competition between replication and repair, or other enzymatic processes, and could also reflect stochasticities at the single-cell level [26]. We found that some of these strains (OV16, D3 and PAT84) had missense mutations in other *dcw* cluster genes in addition to the targeted genes. Transduction of the *ftsA2* and *ftsA3* alleles to the original OV2 strain served to clean the genetic backgrounds but lacked enough resolution to transfer only the targeted mutations. As stated in the results section, the additional mutations found within the *dcw* cluster are likely to be neutral, but these strains have dozens of other missense variants in their genomes, and it is possible that some of them may be contributing to the filamentation phenotypes or that they provided a fitness compensation that facilitated the selection and survival of these division-impaired mutants. It is difficult to extract functional information with this reduced set of four lineages (OV2/OV16/D2/D3, MC1061/VIP205, PAT84 and TOE1). Both the OV2 lineage and PAT84 contain different missense mutations in the *mcrB* (PBP1gamma) and *murA* genes, and there are some other mutations in other genes related to cell wall synthesis and recycling, suggesting a possible compensatory role of these cell wall synthesis pathways (Supplemetary File .

The accumulation of mutations during mutagenesis and subculturing underlines the importance of recognizing that historical laboratory strains and their derivatives may harbour significantly more genetic variation than is captured in the published reference genotypes [27,29]. Unregistered mutations may be confounding factors, potentially leading to the misinterpretation of phenotypic assays or compromising the reproducibility of experimental findings. Therefore, whole-genome sequencing of all strains, and particularly those obtained by mutagenesis, must be adopted as a routine, prerequisite step in repositories of historical strains. This would ensure an accurate and definitive genotypic knowledge for the strains under investigation, which is essential for the reliable interpretation of experimental data.

## Supplementary material

10.1099/mgen.0.001558 Uncited Supplementary Material 1.

## Abbreviations

SNP: single nucleotide polymorphism
